# Cilengitide in newly diagnosed glioblastoma: biomarker expression and outcome

**DOI:** 10.18632/oncotarget.7588

**Published:** 2016-02-22

**Authors:** Michael Weller, Louis Burt Nabors, Thierry Gorlia, Henning Leske, Elisabeth Rushing, Pierre Bady, Christine Hicking, James Perry, Yong-Kil Hong, Patrick Roth, Wolfgang Wick, Simon L. Goodman, Monika E. Hegi, Martin Picard, Holger Moch, Josef Straub, Roger Stupp

**Affiliations:** ^1^ Department of Neurology, University Hospital Zurich and University of Zurich, Zurich, Switzerland; ^2^ University of Alabama at Birmingham, Birmingham, AL, USA; ^3^ EORTC Data Centre, Brussels, Belgium; ^4^ Institute of Neuropathology, University Hospital Zurich, Zurich, Switzerland; ^5^ Department of Education and Research, University of Lausanne, Lausanne, Switzerland; ^6^ SIB Swiss Institute of Bioinformatics, Lausanne, Switzerland; ^7^ Department of Clinical Neurosciences, University Hospital Lausanne, Lausanne, Switzerland; ^8^ Department of Translational and Biomarkers Research, Oncology, Merck KGaA, Darmstadt, Germany; ^9^ Sunnybrook Health Sciences Centre, Toronto, ON, Canada; ^10^ The Catholic University of Korea, Seoul St. Mary's Hospital, Seoul, Korea; ^11^ Neurology Clinic, University of Heidelberg, Heidelberg, Germany; ^12^ Clinical Cooperation Unit (CCU) Neurooncology, German Cancer Consortium (DKTK), German Cancer Research Center (DKFZ), Heidelberg, Germany; ^13^ Institute of Surgical Pathology, University Hospital Zurich, Zurich, Switzerland; ^14^ Department of Oncology, University Hospital Zurich, Zurich, Switzerland

**Keywords:** glioblastoma, integrin, pSmad, TGF-β, biomarker

## Abstract

Integrins αvβ3 and αvβ5 regulate angiogenesis and invasiveness in cancer, potentially by modulating activation of the transforming growth factor (TGF)-β pathway. The randomized phase III CENTRIC and phase II CORE trials explored the integrin inhibitor cilengitide in patients with newly diagnosed glioblastoma with *versus* without O^6^-methylguanine DNA methyltransferase (*MGMT*) promoter methylation. These trials failed to meet their primary endpoints.

Immunohistochemistry was used to assess the levels of the target integrins of cilengitide, αvβ3 and αvβ5 integrins, of αvβ8 and of their putative target, phosphorylation of SMAD2, in tumor tissues from CENTRIC (n=274) and CORE (n=224).

αvβ3 and αvβ5 expression correlated well in tumor and endothelial cells, but showed little association with αvβ8 or pSMAD2 levels. In CENTRIC, there was no interaction between the biomarkers and treatment for prediction of outcome. In CORE, higher αvβ3 levels in tumor cells were associated with improved progression-free survival by central review and with improved overall survival in patients treated with cilengitide.

Integrins αvβ3, αvβ5 and αvβ8 are differentially expressed in glioblastoma. Integrin levels do not correlate with the activation level of the canonical TGF-β pathway. αvβ3 integrin expression may predict benefit from integrin inhibition in patients with glioblastoma lacking *MGMT* promoter methylation.

## INTRODUCTION

Integrins are a family of 24 heterodimeric cell surface receptors that participate in signal transduction during many cellular processes. They are also involved in cellular communication with the extracellular matrix, e.g. during adhesion, motility, migration, invasion and angiogenesis. Their abundant expression in tumor-associated endothelial cells [1, 2] and presumed biological roles led to integrins αvβ3 and αvβ5 being identified and validated as therapeutic targets in glioblastoma in preclinical models [3, 4]. These data supported the clinical development program for the pentapeptide, first-in-class integrin inhibitor, cilengitide [5, 6]. In phase I, dose-limiting toxicity was not seen at doses up to 2400 mg/m^2^, whereas clinical activity was seen at both low and high levels [7]. A randomized phase II trial in recurrent glioblastoma comparing two different doses of cilengitide noted a moderate radiological response rate, interpreted to reflect biological activity, and a trend towards better outcome with the higher dose of cilengitide [8]. Improved outcome at higher dose was also observed in a randomized phase II trial in the newly diagnosed setting in combination with the standard of care, temozolomide chemoradiotherapy (TMZ/RT→TMZ) [9]. An earlier uncontrolled phase II trial indicated preferential benefit from cilengitide in newly diagnosed glioblastoma patients with, as opposed to without, O^6^-methylguanine DNA methyltransferase (*MGMT*) promoter methylation [10].

Based on these data, separate trials were designed for patients with (“CENTRIC”) and without (“CORE”) *MGMT* promoter methylation. The phase III CENTRIC trial was designed to verify the activity of cilengitide in newly diagnosed patients with *MGMT* promoter methylation. The exploratory phase II CORE trial explored, in addition, whether intensified the dose of cilengitide during radiotherapy might provide a signal of activity in patients with tumors lacking *MGMT* promoter methylation, too. Neither trial demonstrated biological activity of cilengitide defined by the primary endpoints of the trials [11, 12], resulting in the discontinuation of the clinical development of cilengitide.

Detailed analysis of the expression of the target integrins of cilengitide, αvβ3 and αvβ5 in tumor and endothelial cells, might result in a better understanding of these disappointing trial results. Unfortunately, tumor tissues were not systematically collected in the earlier trials, and appropriate antibodies have only recently been generated [13].

While αvβ3 and αvβ5 expression in glioblastoma have been related mainly to angiogenesis, αvβ8 has been attributed roles in migration and invasion [14-16]. Among the multiple effects of integrin signaling, we have recently delineated how the target integrins of cilengitide, αvβ3 and αvβ5, as well as αvβ8 integrin, may control activity of the transforming growth factor (TGF)-β pathway [17-19], which has been linked to the malignant phenotype of glioblastoma. Specifically, we observed that either exposure to cilengitide or gene silencing of αvβ3, αvβ5 or αvβ8, or neutralizing antibodies to these integrins reduced (TGF)-β_1/2_ mRNA expression, protein release and pSMAD2 phosphorylation, a surrogate marker of canonical TGF-β pathway activation [20], in glioma cells [17]. Conversely, integrin αvβ3 expression had previously been reported to be induced by TGF-β [21], potentially constituting a positive feedback loop. These data indicated that pSmad2 levels could serve as a biomarker to identify integrin signaling-dependent tumors. While the prognostic role of elevated pSMAD2 levels has remained controversial [20, 22], TGF-β itself is also a candidate therapeutic target in glioblastoma [23]. Accordingly, here we studied integrin expression profiles in tumors of patients enrolled in the CENTRIC and CORE trials and explored whether these expression profiles were related to levels of pSMAD2 and outcome.

## RESULTS

### Tumor and patient characteristics

We studied the levels of integrins αvβ3, αvβ5 and αvβ8 and of pSMAD2 by immunocytochemistry in tissues obtained at study entry from patients randomized into the CENTRIC or CORE trials. Tissue samples from 498 patients were analyzed, representing 61% of the patient cohorts. For 39% of the patients, no or insufficient tumor tissue was submitted or available for ancillary biological investigations. Samples were received from 106 and 52 centers in CENTRIC and CORE, respectively, whereof 35 centers were in common. Patient characteristics, treatment received and outcome by trial and in the biomarker cohort are summarized in Table 1. Patients in all groups received a median of 6 cycles of TMZ. That PFS in CORE is still only 6 months from randomization, can be explained by the recommendation to consider the possibility of pseudoprogression in the adjuvant treatment phase and not to stop adjuvant TMZ too early unless there was unequivocal PD. There was no significant outcome difference between the patients in the biomarker cohort and those where biomarkers were not evaluated (Supplementary Table S1).

**Table 1:** Summary of patient characteristics, treatment delivery and outcome

	CENTRIC All Patients N=545	CENTRIC Biomarker Cohort n=274	CORE All Patients N=265	CORE Biomarker Cohort n=224
**Age at baseline**				
Median (years)	57.9	58.8	56.2	56.6
Range (years)	21.7 - 81.0	21.7-81.0	20.8 - 77.5	20.8 - 76.5
**Gender**				
Male	291 (53.4)	148 (54.0%)	155 (58.5)	131 (58.5)
Female	254 (46.6)	126(46.0%)	110 (41.5)	93 (41.5)
**Histological subtype**				
**Glioblastoma**	496 (91.0)	248 (90.5)	251 (94.7)	211 (94.2)
**Gliosarcoma**	21 (3.9)	10 (3.6)	10 (3.8)	9 (4.0)
**Giant cell**	17 (3.1)	12 (4.4)	3 (1.1)	3 (1.3)
**Other**	11 (2.0)	4 (1.5)	1 (0.4)	1 (0.4)
**ECOG Performance Status at baseline**				
PS 0	309 (56.7)	163 (59.5)	131 (49.4)	111 (49.6)
PS 1	236 (43.3)	111 (40.5)	132 (49.8)	111 (49.6)
No data	0 (0.0)	0 (0.0)	2 (0.8)	2 (0.9)
**Surgery**				
Subtotal resection (partial/biopsy)	274 (50.3)	128 (46.7)	128 (48.3)	102 (45.5)
Gross total resection	269 (49.4)	144 (52.6)	136 (51.3)	121 (54.0)
No data	2 (0.4)	2 (0.7)	1 (0.4)	1 (0.4)
**Treatment received**				
	TMZ (n=273)	TMZ (n=137)	TMZ (n=89)	TMZ (n=71)
Received study intervention	258 (94.5)	130 (94.9)	85(95.5)	68 (95.8)
Started RTX	256 (93.8)	129 (94.2)	85(95.5)	68 (95.8)
Started maintenance TMZ	211 (77.3)	106 (77.4)	71(80.0)	58 (81.7)
Number of TMZ maintenance cycles				
Median	6	6	6	6
Range	1-32	1-21	1-11	1-8
	Cilengitide (n=272)	Cilengitide (n=137)	Cilengitide (n=176)	Cilengitide (n=153)
Received study intervention	263 (96.7)	133 (97.0)	170(96.6)	147(96.1)
Started Pre-RTX phase	259 (95.2)	131 (95.6)	168(95.5)	146(95.4)
Started RTX phase	260 (95.6)	131 (95.6)	168(95.5)	146(95.4)
Started maintenance TMZ	221 (81.3)	111 (81.0)	144(81.8)	125(81.7)
Number of TMZ maintenance cycles				
Median	6	6	6	6
Range	1-21	1-21	1-19	1-19
Started cilengitide monotherapy phase	168 (61.8)	77 (56.2)	85(48.3)	74(48.4)
Total number of cilengitide infusions				
Median	90	72	69	69
Range	1-388	1-317	2-224	2-224
**Outcome**				
Median PFS (months, 95% CI)	12.3 (10.6, 13.6)	12.1 (10.4,13.6)	6.2 (5.9, 7.7)	6.3 (5.9, 7.7)
Median OS (months, 95% CI)	26.3 (24.4, 29.3)	25.4 (23.3,30.9)	14.4 (13.4, 15.6)	14.1 (12.9, 15.5)

### Integrin and pSMAD2 staining patterns

**Figure 1:**
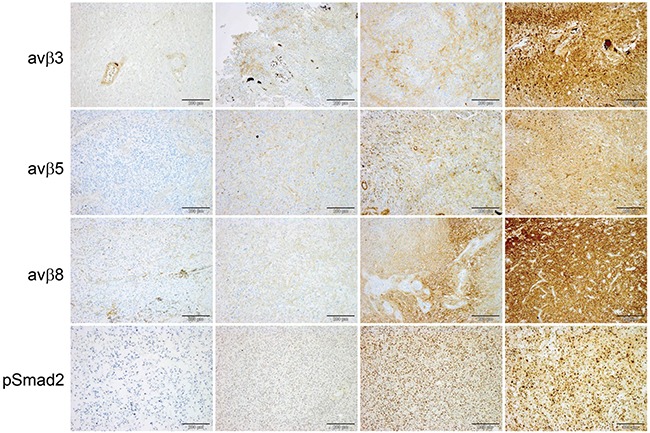
Immunohistochemical assessment of integrins and pSmad2 in glioblastoma Representative sections immunostained for αvβ3, αvβ5, αvβ8 and pSMAD2. Negative staining of tumor tissue with immunolabeled vasculature as internal positive control (left column), H score (tumor) < 100 second column, H score (tumor) 101-200 third column, H score (tumor) >200 (right column), size bars correspond to 200 μm.

**Figure 2:**
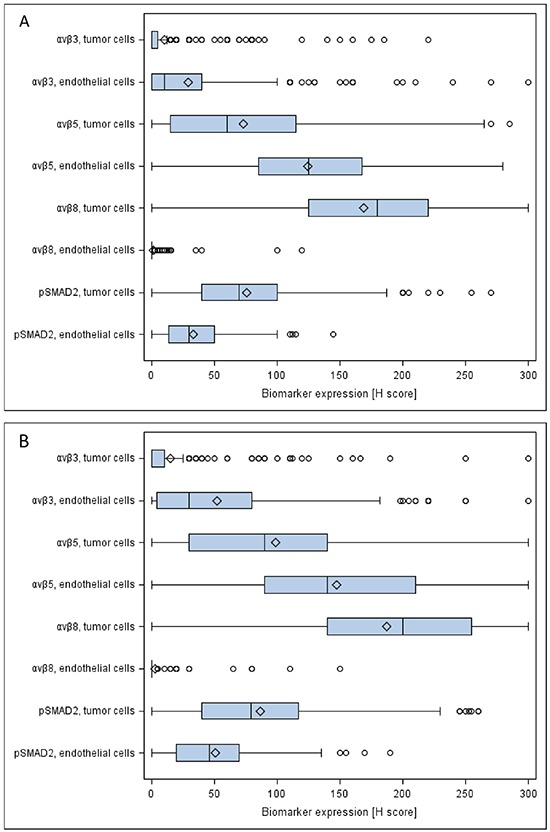
Quantitative assessment of immunohistochemistry data Boxplots of biomarker expression in the CENTRIC **A.** and CORE **B.** biomarker cohorts are depicted. The boxes represent the interquartile range split by the medians. Diamonds represent the means. Lines extending horizontally from the boxes indicate variability outside the range (lowest value within 1.5 IQR of Q1, and highest value within 1.5 IQR of Q3). Outliers beyond 1.5 IQR are plotted as individual points.

**Figure 3:**
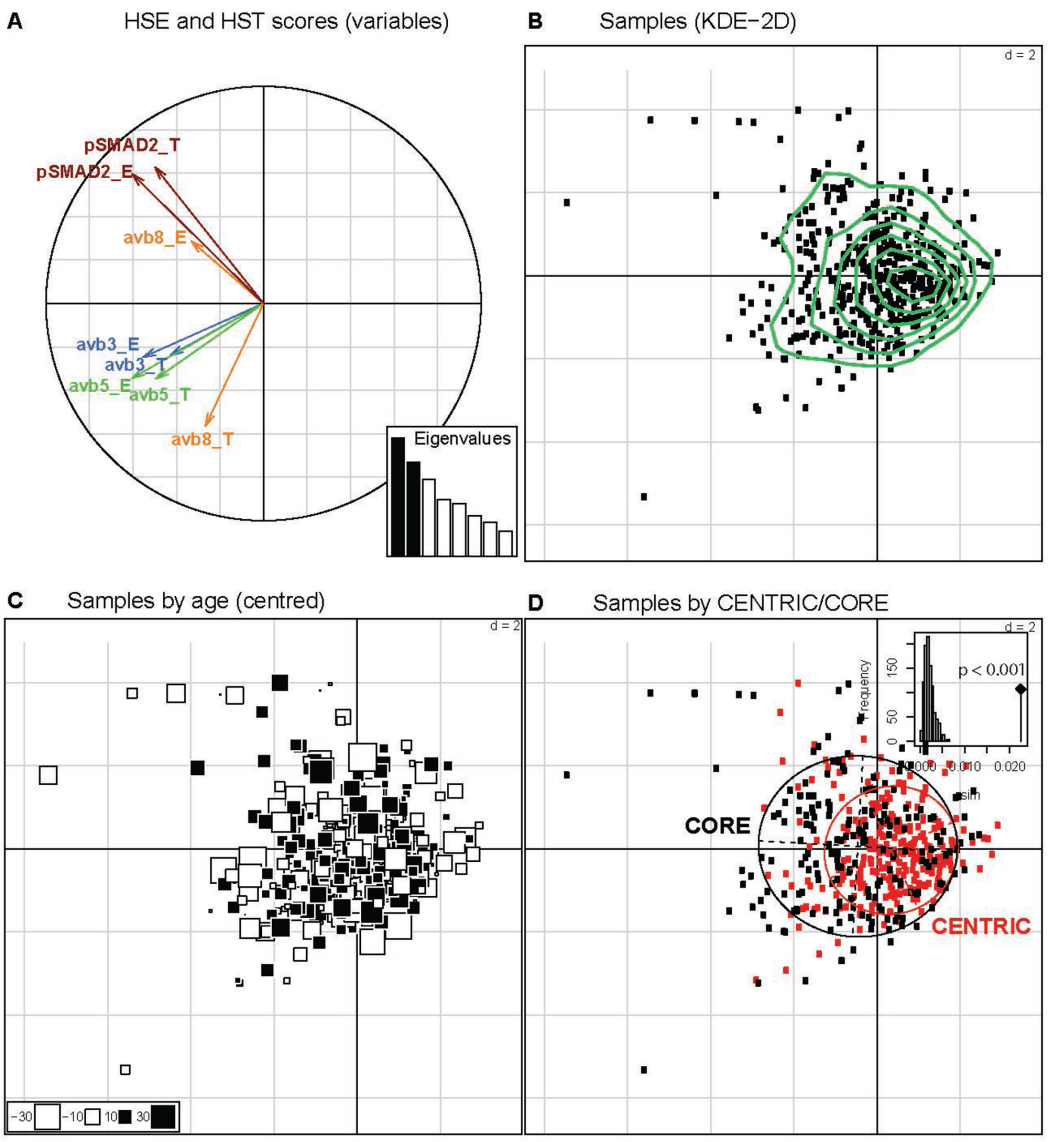
Principal component analysis (PCA) of biomarker analyses **A.** The histoscores of the markers for tumor (HST) and endothelial cells (HSE) are represented on the first vectorial plan of the PCA. The two first distinct eigenvalues (Histogram of Eigenvalues, in black) explain 42.6% of the total variation. **B.** The patient samples are projected onto the two first axes of the PCA and patterns were explored by Kernel Density Estimation (KDE) in these two dimensions (green curves). No indications for marker driven subgroups are observed. **C.** Each patient sample is represented by a square, with proportional size to the distance to the mean age (55.3 years). The white and black squares identify the patients with age inferior or superior to the mean age, respectively. **D.** The impact of the study (CENRTIC/CORE) was investigated on the two first axes visualized by the inertia ellipses for CENTRIC (red) and CORE (black). A significant difference is observed (P < 0.001, between-group permutation tests) illustrated by a histogram, where the observed value is given by a black vertical line.

The expression of the integrins within most tumor samples was heterogeneous. Staining was localized to the cytoplasm with sparing of the nuclei and without membranous accentuation. Representative staining patterns illustrating the H scores are depicted in Figure 1. Antigen expression was evaluated separately in the tumor and endothelial compartments. The quantitative assessments are summarized in Table 2 and Figure 2. In CENTRIC, αvβ3 levels in tumor cells correlated weakly with αvβ3 in endothelial cells (SSC=0.26, p<0.0001), with αvβ5 in tumor cells (SSC=0.18, p=0.002) and with αvβ5 levels in endothelial cells (SSC=0.16, p=0.006). αvβ5 levels in tumor and endothelial cells were also weakly correlated (SSC=0.17, p=0.003) whereas pSMAD2 levels in both compartments showed strong correlation (SSC=0.50, p<0.0001). In CORE, αvβ3 levels in tumor cells weakly correlated with αvβ3 levels in endothelial cells (SSC=0.26, p<0.001) and with αvβ5 levels in endothelial cells (SSC=0.29, p<0.001). αvβ5 levels in tumor and endothelial cells were weakly correlated (SSC=0.29, p<0.001). αvβ5 levels in endothelial cells correlated weakly with αvβ8 in tumor cells (SSC=0.21, p=0.002). There was good correlation between pSMAD2 levels in both compartments (SSC=0.55, p<0.001) (Supplementary Table S2). Investigation of the relationship among the different markers by PCA illustrated in Figure 3A underlines the correlations detailed above. It does not support a direct relationship between the integrins and pSMAD2 levels as measured by immunohistochemistry. The markers analyzed do not segregate the tumors into different subgroups (Figure 3B). Further, exploration of the major sources of variation among tumors did not indicate any difference by gender (p=0.088, not shown), or age (Figure 3C, p=0.380), but a significant difference between the two studies, CENTRIC and CORE (p=0.001) was observed (Figure 3D). However, the analysis of the variation fraction revealed that the variable “center” explained 40% of the total variation (between-group ratio=0.403, p-value < 0.001), and only 2% were attributed to differences between the studies (between-group ratio=0.022, p-value< 0.001). Nevertheless, the overall structure of the relationships among markers between CORE and CENTRIC, when analyzed separately, is preserved (not shown).

**Table 2:** Quantitative assessment of immunohistochemistry data

	CENTRIC biomarker cohort n=274*	CORE biomarker cohort n=224*
αvβ3 tumor cells		
Median	0	0
Range	0–220	0–300
N	294	241
αvβ3 endothelial cells		
Median	10	30
Range	0-300	0-300
N	294	241
αvβ5 tumor cells		
Median	60	90
Range	0-285	0-300
N	294	237
αvβ5 endothelial cells		
Median	125	140
Range	0-280	0-300
N	292	236
αvβ8 tumor cells		
Median	180	200
Range	0-300	0-300
N	283	231
αvβ8 endothelial cells		
Median	0	0
Range	0-120	0-150
N	283	231
pSMAD2 tumor cells		
Median	70	79
Range	0-270	0-260
N	281	227
pSMAD2 endothelial cells		
Median	30	46
Range	0-145	0-190
N	281	227

* Note that n in the table may be higher for individual markers since the biomarker cohorts were defined as patients with tumors where all markers were assessed.

### Clinical pathological correlations

**Figure 4:**
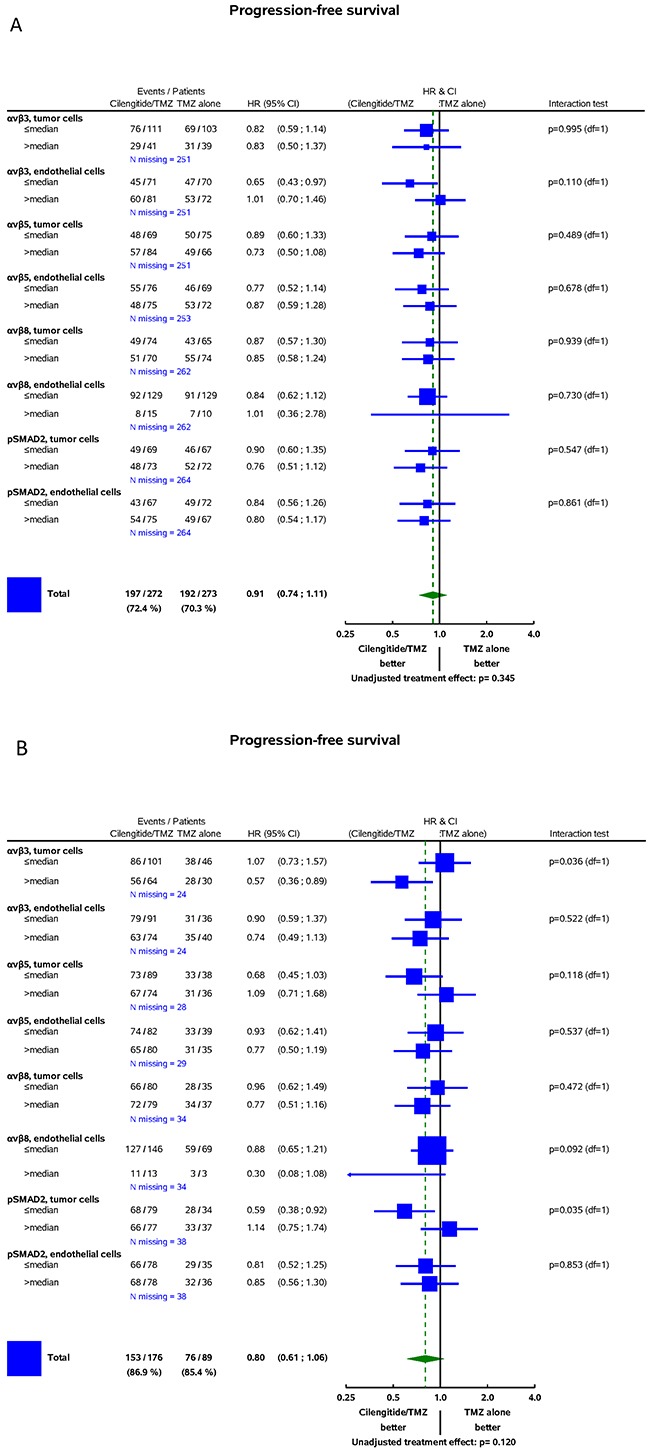
Forest plots: predictive value of biomarkers for the efficacy of cilengitide for PFS assessed by central review **A.** CENTRIC. **B.** CORE. On the left-hand, the integrin subgroups with numbers of events/sample size by treatment arm, number of missing data, and hazard ratios (HR) with 95% confidence intervals are shown. The vertical line represents the absence of differential effects between the two treatments, i.e., if for an integrin subgroup, the 95% confidence intervals overlap with this line, it indicates that treatment effects are not different. The square represents the Cilengitide/TMZ hazard ratio in the integrin subgroup. The area of each square is proportional to the number of events. The diamond indicates a differential effect of treatment in the whole cohort. Diamond overlapping the vertical lines indicates in-significantly different treatment effects at 5% significance. On the right-hand, interaction tests are presented. They assess the significance of a differential treatment effect between two integrin subgroups, i.e., tests have one degree of freedom (df=1).

**Figure 5:**
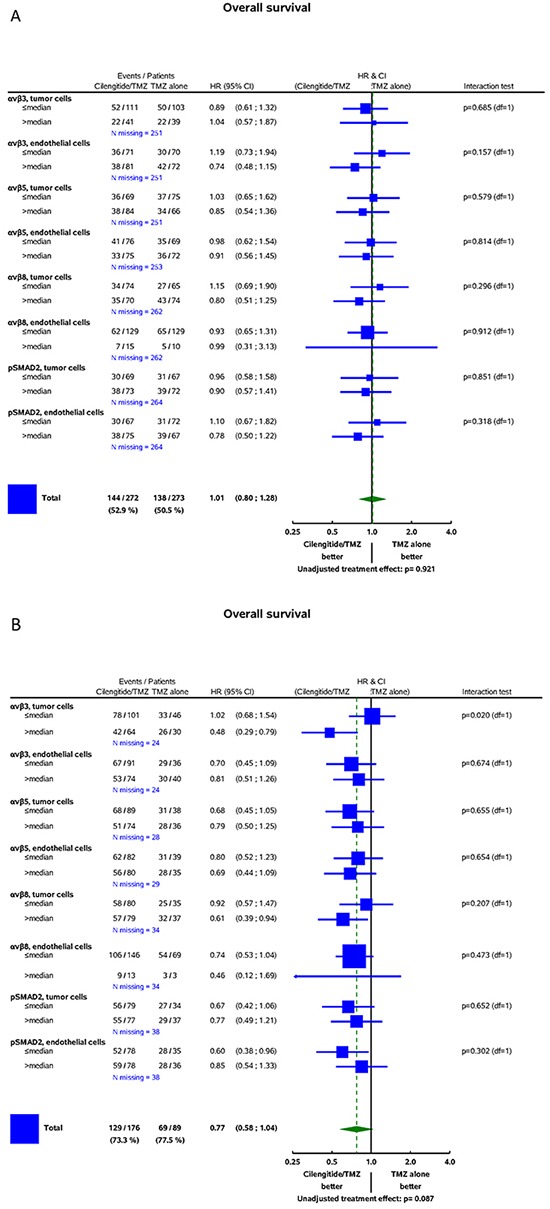
Forest plots: predictive value of biomarkers for the efficacy of cilengitide for OS **A.** CENTRIC **B.** CORE (for detailed explanations, see Figure 4).

In the CENTRIC cohort, there was no significant interaction between the biomarkers and treatment for the prediction of PFS determined by central review (Figure 4A) or investigator assessment (Supplementary Figure S1A) or for the prediction of OS (Figure 5A). In contrast, in CORE, higher αvβ3 levels in tumor cells were associated with improved PFS by central review (Figure 4B, p=0.036) and improved OS (Figure 5B, p=0.02) in patients treated with cilengitide. This effect persisted when analysed stratified for prognostic factors, including age, RPA score, extent of surgery, MMSE, or ECOG PS (data on shown). However, the PFS effect was not confirmed when exploring investigator-assessed PFS (investigator PFS interaction test p=0.345, IRC PFS p=0.036) (Supplementary Figure S1B).

In order to maximize the statistical power, the univariate prognostic value of the biomarkers was assessed in the whole cohort. No significant association of integrin expression in tumor or endothelial cells with outcome was revealed in either trial cohort. Similarly, no prognostic role for pSMAD2 levels became apparent (Supplementary Table S3 and S4).

## DISCUSSION

The main goal of this study was to explore whether the expression patterns of the putative target integrins of cilengitide, αvβ3 and aβ5, might shed light on the results of the CENTRIC and CORE clinical trials. We find that the target integrins are differentially expressed in tumor and endothelial cells within glioblastomas (Figures 1 and 2, Table 2). In contrast to previous analyses of smaller, non-clinical trial cohorts, [24] integrin expression was neither prognostic in the CENTRIC nor in the CORE biomarker cohort. The differential expression of target integrins either in tumor or in endothelial cells was unrelated to outcome with cilengitide treatment in the CENTRIC trial. In contrast, higher αvβ3 levels in tumor cells were associated with improved PFS by central review and with improved OS in cilengitide-treated patients in the CORE cohort (Figure 4B and 5B). Patient cohorts from both trials were analysed separately because of the powerful prognostic role of *MGMT* promoter methylation, the biomarker that determined patient enrolment in either CENTRIC or CORE.

The levels of αvβ3 and αvβ5 correlated weakly, both in tumor and endothelial cells, suggesting a common upstream regulatory pathway regulating integrin expression in both compartments in glioblastoma. In contrast, the levels of αvβ3 and αvβ5 showed almost no relation to the levels of αvβ8. While αvβ8 is not a target of cilengitide, it may be involved in the activation of latent TGF-β [17, 25]. Importantly, there is no surrogate biomarker reflecting integrin activity as opposed to mere expression which may be heterogeneous within a tumor [26], and defining a biomarker of integrin pathway activation may hαve been more informative, e.g., levels of focal adhesion kinase or other focal adhesion-associated proteins. Importantly, however, the present analysis suggests that enrichment for patients with tumors with high levels of target integrin expression would not hαve altered the negative outcome at least in the CENTRIC trial.

A second goal of this study was to verify whether the involvement of αv integrin in controlling TGF-β pathway activity [17] is reflected by correlated integrin expression and Smad2 phosphorylation, a marker for TGF-β pathway activity [20]. This was not confirmed in either trial cohort (Figure 3; Supplementary Table S2), suggesting that expression of integrins alone is not an adequate surrogate marker for integrin activity *in situ*. Alternatively, pSMAD2 levels in glioblastoma and the TGF-β pathway may not be controlled by αv integrins to a relevant extent. Interestingly, however, there was strong correlation between pSMAD2 levels in tumor and in endothelial cells, suggesting that both compartments are equally responsive to TGF-β activity in the tumor microenvironment. Thus, escape from the inhibitory signaling activity of TGF-β as seen in other cancers, e.g., colon cancer, is not required in glioblastoma, at least not at the level of Smad2 canonical signaling.

In summary, the expression patterns of the integrin targets of cilengitide, αvβ3 and αvβ5, did not provide prognostic information and did not reveal glioblastoma patient populations that were more or less responsive to cilengitide in the phase III CENTRIC trial. The weak association with improved outcome with cilengitide in patients with glioblastoma lacking *MGMT* promoter methylation in the phase II CORE trial may justify patient enrichment based on αvβ3 expression in tumor cells in future trials. Yet, agents more potent than cilengitide will be needed to explore whether integrins are a relevant target in glioblastoma, and indicators of integrin pathway activation may be superior biomarkers over integrin expression levels for patient enrichment.

## MATERIALS AND METHODS

### Patients

We examined tumor tissues obtained at study entry of patients with newly diagnosed glioblastoma enrolled in the CENTRIC (NCT00689221) and CORE (NCT00813943) trials [11, 12]. Accurate immunohistochemical detection of antigen in tissue was based on procedures published by Vogetseder et al. [27] which analyzed integrin expression in a TMA containing 152 cores of non-neoplastic tissue in our laboratory. The biomarker cohorts were defined as patients in each trial where all markers could be assessed. All patients provided written informed consent for the clinical investigation and correlative science reported here. The protocols were approved by the local ethics committees or institutional review boards, and appropriate regulatory authorities.

### Immunohistochemistry

Four μm sections of tumor tissue from paraffin-embedded blocks were deparaffinized and immunostained for the expression of integrin αvβ3 (clone EM22703, 5 μg ml^−1^), αvβ5 (clone EM09902, 1 μg ml^−1^) and αvβ8 (clone EM13309, 1.3 μg ml^−1^) [13, 17, 27] as well as pSMAD2 expression (Cell Signaling clone 138D4, 1:200), according to the Ventana protocols (Ventana Medical System). The semiquantitative expression level and area of staining on each section for αvβ3, αvβ5 and αvβ8 integrins and for pSMAD2 were assessed by two neuropathologists, independently in glioma cells and endothelial cells within the tumor using the semiquantitative histoscore (H-Score) method [28, 29]. Briefly, staining intensity is scored as absent (0), mild (1), moderate (2) or strong (3) expression. The staining intensity value is multiplied by the percentage of cells showing each grade of positivity, for a maximum total score of 300. The neuropathologists were blinded with regard to patient allocation to treatment arm and outcome. The concordance between the two neuropathologists was 100% since any differences in the initial evaluation, which were always minor, were sorted out until a consensus was reached. Also noteworthy is the fact that even though the absolute percentages of the various components differed somewhat among the two pathologists, the H-score proved to be the same.

### Statistical analysis

Continuous variables (including biomarkers) were presented using median and range (minimum, maximum). Boxplots were drawn to visualize biomarker distributions. Frequency tables were tabulated (by whole trial and biomarker cohort) for all categorical variables. Spearman Correlation Coefficients (SCC) were computed to quantify the relationships between biomarkers. Correlations with p values less than 1% are summarized. SSC less than or equal to 0.3 was considered a weak correlation. SCC between 0.3 and 0.49 was considered fair correlation and SSC equal to or above 0.5 a good correlation.

Principal component analysis (PCA) was performed to explore and to illustrate the correlation pattern among the markers. A Monte-Carlo test on between-group inertia (global test) based on the percentage of explained variation was used to test the overall difference between gender, centers and CENTRIC and CORE trials [30]. The PCA, between-group test analyses and graphic representations were performed using R package ade4 [31].

For progression-free survival (PFS) and overall survival (OS), Kaplan Meier curves were computed for each biomarker split by their median in each trial. Score tests obtained from univariate Cox regression models were used to assess the prognostic value of each biomarker in the biomarker cohort. Predictive value for treatment efficacy was assessed by Cox regression including treatment (TMZ/RT→TMZ versus TMZ/RT→TMZ+Cilengitide), biomarker (≤ median versus > median) and treatment by biomarker interaction score tests. All outcome analyses were exploratory and performed without adjustment for multiplicity at 5% significance, and outcome parameters (i.e. medians, hazard ratios) were presented with 95% confidence intervals. In the CORE trial, data of both cilengitide arms pooled.

## SUPPLEMENTARY FIGURES AND TABLES








