# Supplementary material for: Cilengitide in newly diagnosed glioblastoma: biomarker expression and outcome
Source: Oncotarget. 2016 Feb 22;7(12):15018–32. doi: 10.18632/oncotarget.7588 (PMC4924768; doi:10.18632/oncotarget.7588)
Supplement: Supplementary file 1 [file oncotarget-07-15018-s001.pdf]

SUPPLEMENTARY FIGURES AND TABLES

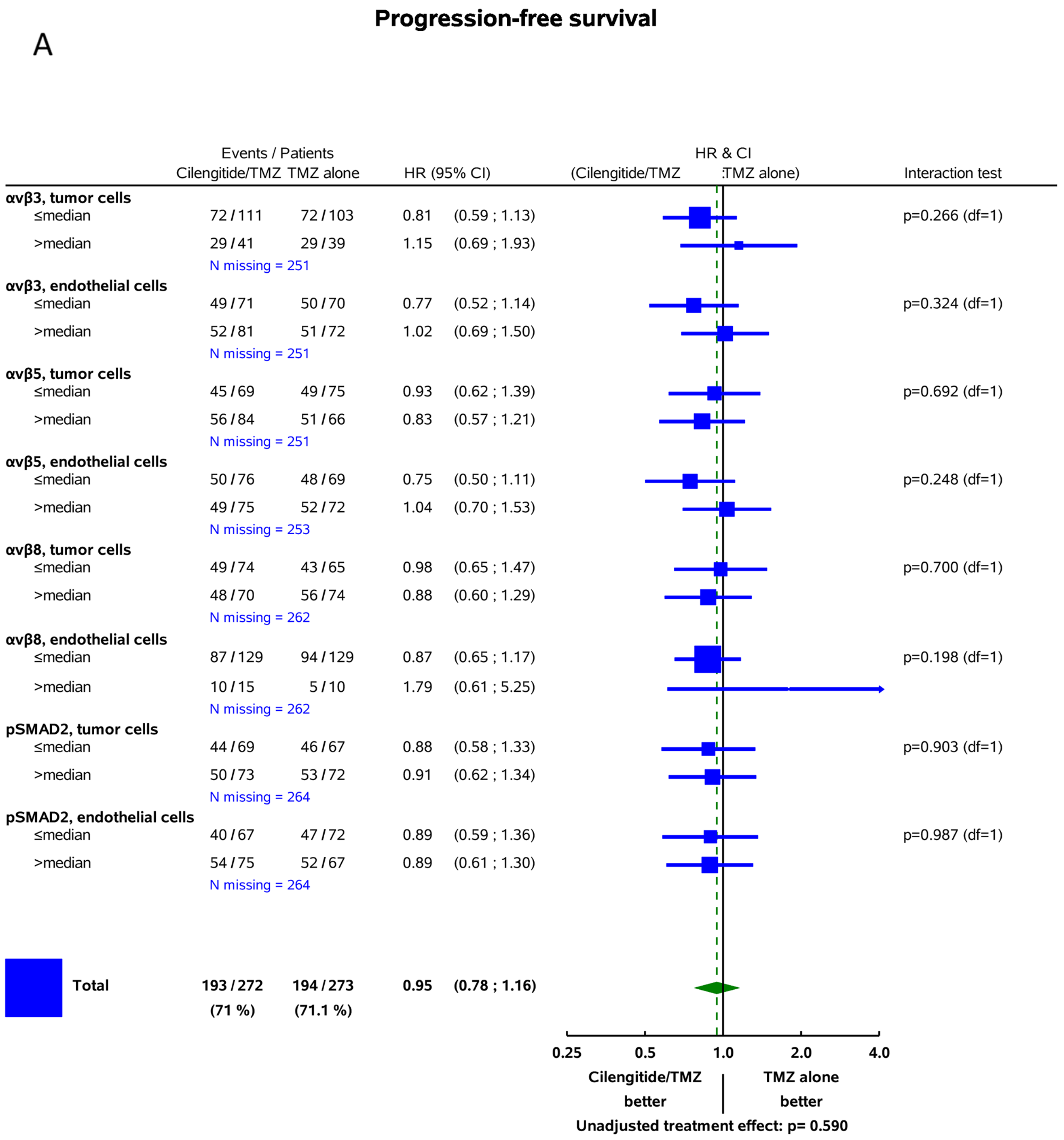

Supplementary Figure 1: Forest plots: predictive value of biomarkers for the efficacy of cilengitide for investigator-assessed PFS. A. CENTRIC (*continued*)

Progression-free survival

B

	Events / Patients Cilengitide/TMZ	TMZ alone	HR (95% CI)		Interaction test
**αvβ3, tumor cells**					
≤median	84 / 101	39 / 46	0.86	(0.59 ; 1.26)	p=0.345 (df=1)
>median	56 / 64	28 / 30	0.65	(0.41 ; 1.02)	
	N missing = 24				
**αvβ3, endothelial cells**					
≤median	77 / 91	29 / 36	0.80	(0.52 ; 1.23)	p=0.732 (df=1)
>median	63 / 74	38 / 40	0.72	(0.48 ; 1.09)	
	N missing = 24				
**αvβ5, tumor cells**					
≤median	71 / 89	34 / 38	0.54	(0.36 ; 0.82)	p=0.019 (df=1)
>median	67 / 74	31 / 36	1.11	(0.72 ; 1.70)	
	N missing = 28				
**αvβ5, endothelial cells**					
≤median	69 / 82	32 / 39	0.78	(0.51 ; 1.18)	p=0.982 (df=1)
>median	68 / 80	33 / 35	0.77	(0.51 ; 1.17)	
	N missing = 29				
**αvβ8, tumor cells**					
≤median	66 / 80	28 / 35	0.83	(0.53 ; 1.29)	p=0.807 (df=1)
>median	69 / 79	35 / 37	0.77	(0.51 ; 1.16)	
	N missing = 34				
**αvβ8, endothelial cells**					
≤median	125 / 146	60 / 69	0.80	(0.59 ; 1.09)	p=0.637 (df=1)
>median	10 / 13	3 / 3	0.58	(0.16 ; 2.13)	
	N missing = 34				
**pSMAD2, tumor cells**					
≤median	66 / 79	29 / 34	0.56	(0.36 ; 0.87)	p=0.037 (df=1)
>median	65 / 77	33 / 37	1.06	(0.70 ; 1.62)	
	N missing = 38				
**pSMAD2, endothelial cells**					
≤median	63 / 78	28 / 35	0.69	(0.44 ; 1.09)	p=0.492 (df=1)
>median	68 / 78	34 / 36	0.86	(0.57 ; 1.30)	
	N missing = 38				
**Total**	**151 / 176 (85.8 %)**	**77 / 89 (86.5 %)**	**0.75**	**(0.57 ; 0.99)**	

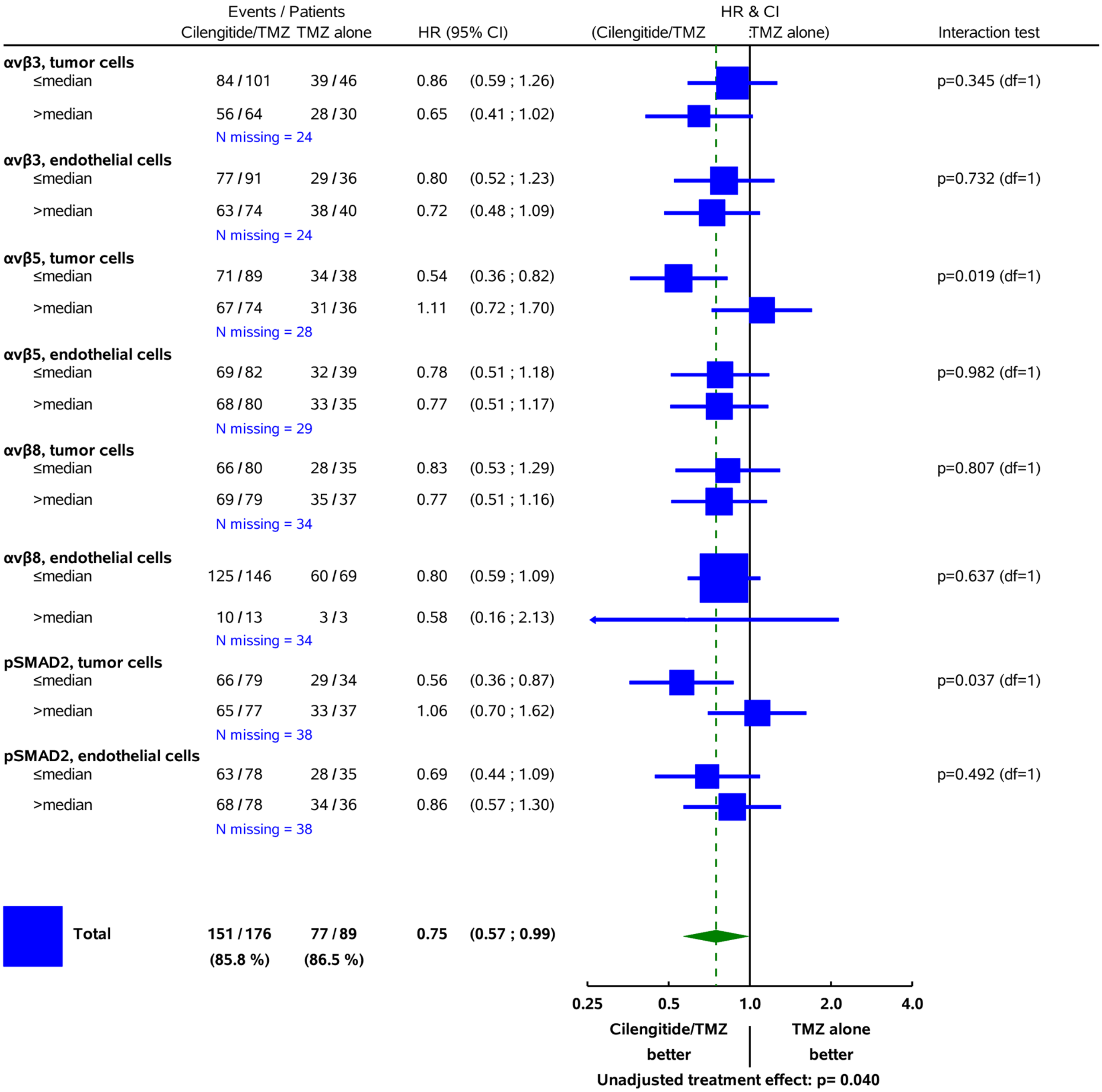

Supplementary Figure 1: (*continued*) Forest plots: predictive value of biomarkers for the efficacy of cilengitide for investigator-assessed PFS. B. CORE. On the left-hand, the integrin subgroups with numbers of events/sample size by treatment arm, number of missing data, and hazard ratios (HR) with 95% confidence intervals are shown. The vertical line represents the absence of differential effects between the two treatments, i.e., if for an integrin subgroup, the 95% confidence intervals overlap with this line, it indicates that treatment effects are not different. The square represents the Cilengitide/TMZ hazard ratio in the integrin subgroup. The area of each square is proportional to the number of events. The diamond indicates a differential effect of treatment in the whole cohort. Diamond overlapping the vertical lines indicates in-significantly different treatment effects at 5% significance. On the right-hand, interaction tests are presented. They assess the significance of a differential treatment effect between two integrin subgroups, i.e., tests have one degree of freedom (df=1).

Supplementary Table 1: Outcome in the CENTRIC and CORE clinical trials: patients without or with incomplete biomarker data compared to patients in the biomarker cohort

	Progression-free survival				Overall survival			
	**Patients/ events**	**Median (95% CI)**	**Hazard ratio**	**p value**	**Patients/ events**	**Median (95% CI)**	**Hazard ratio**	**p value**
**CENTRIC**								
No or incomplete biomarker data	271/199	13.0 (10.1, 15.1)	1.0	0.57	271/148	27.1 (24.8, 33.7)	1.0	0.45
Biomarker cohort	274/188	12.1 (10.4, 13.6)	1.1 (0.9, 1.3)		274/134	25.4 (23.3, 30.9)	1.1 (0.9, 1.4)	
**CORE**								
No or incomplete biomarker data	41/37	6.0 (4.0, 9.9)	1.0	0.86	41/32	16.6 (12.0, 20.5)	1.0	0.46
Biomarker cohort	224/191	6.3 (5.9, 7.7)	1.0 (0.7, 1.5)		224/166	14.1 (12.9, 15.4)	1.2 (0.8, 1.7)	

Supplementary Table 2: Correlation of biomarker staining results

(See Supplementary File 1)

Supplementary Table 3: Association of integrin and pSMAD2 levels with PFS

(See Supplementary File 2)

Supplementary Table 4: Association of integrin and pSMAD2 levels with OS

(See Supplementary File 3)
