# Supplementary material for: Cilengitide in newly diagnosed glioblastoma: biomarker expression and outcome
Source: Oncotarget. 2016 Feb 22;7(12):15018–32. doi: 10.18632/oncotarget.7588 (PMC4924768; doi:10.18632/oncotarget.7588)
Supplement: Supplementary file 2 [file oncotarget-07-15018-s002.doc]

Supplementary Table 2. Correlation of biomarker staining results.

**CENTRIC**

|  | | | | | | | | |
| --- | --- | --- | --- | --- | --- | --- | --- | --- |
|  | **αvβ3**  **tumor cells** | **αvβ3**  **endothelial cells** | **αvβ5**  **tumor cells** | **αvβ5**  **endothelial cells** | **αvβ8**  **Tumor cells** | **αvβ8**  **endothelial cells** | **pSMAD2**  **tumor cells** | **pSMAD2**  **endothelial cells** |
| **αvβ3**  **tumor cells** |  | 0.26 <.0001 294 | 0.18 0.002 292 | 0.16 0.006 290 | 0.02 0.79 281 | -0.01 0.81 281 | -0.01 0.89 279 | 0.06 0.30 279 |
| **αvβ3**  **endothelial cells** |  |  | -0.06 0.30 292 | 0.09 0.11 290 | 0.25 <.0001 281 | -0.05 0.44 281 | -0.11 0.07 279 | 0.14 0.02 279 |
| **αvβ5**  **tumor cells** |  |  |  | 0.17 0.003 292 | 0.13 0.029 282 | -0.04 0.55 282 | 0.15 0.011 280 | 0.13 0.04 280 |
| **αvβ5**  **endothelial cells** |  |  |  |  | 0.15 0.01 280 | 0.004 0.95 280 | 0.05 0.44 279 | 0.05 0.41 279 |
| **αvβ8**  **tumor cells** |  |  |  |  |  | -0.13 0.03 283 | 0.08 0.21 278 | 0.09 0.15 278 |
| **αvβ8**  **endothelial cells** |  |  |  |  |  |  | 0.05 0.42 278 | 0.04 0.46 278 |
| **pSMAD2**  **tumor cells** |  |  |  |  |  |  |  | 0.50 <.0001 281 |

CORE

|  | | | | | | | | |
| --- | --- | --- | --- | --- | --- | --- | --- | --- |
|  | **αvβ3**  **tumor cells** | **αvβ3**  **endothelial cells** | **αvβ5**  **tumor cells** | **αvβ5**  **endothelial cells** | **αvβ8**  **Tumor cells** | **αvβ8**  **endothelial cells** | **pSMAD2**  **tumor cells** | **pSMAD2**  **endothelial cells** |
| **αvβ3**  **tumor cells** |  | 0.26 <.0001 241 | 0.14 0.037 236 | 0.07 0.26 235 | 0.001 0.99 230 | -0.04 0.56 230 | -0.14 0.042 226 | -0.07 0.27 226 |
| **αvβ3**  **endothelial cells** |  |  | 0.08 0.24 236 | 0.29 <.0001 235 | 0.12 0.079 230 | 0.06 0.37 230 | -0.07 0.30 226 | 0.10 0.12 226 |
| **αvβ5**  **tumor cells** |  |  |  | 0.29 <.0001 236 | 0.17 0.01 230 | -0.04 0.52 230 | 0.07 0.28 226 | 0.01 0.86 226 |
| **αvβ5**  **endothelial cells** |  |  |  |  | 0.21 0.002 229 | 0.10 0.14 229 | 0.04 0.56 226 | 0.06 0.39 226 |
| **αvβ8**  **tumor cells** |  |  |  |  |  | -0.12 0.075 231 | -0.08 0.23 226 | -0.04 0.52 226 |
| **αvβ8**  **endothelial cells** |  |  |  |  |  |  | 0.15 0.022 226 | 0.15 0.026 226 |
| **pSMAD2**  **tumor cells** |  |  |  |  |  |  |  | 0.55 <.0001 227 |

Note: In each cell, first line corresponds to Spearman Correlation Coefficients (SSC), second line to p-value under null hypothesis of no correlation (SSC=0), third line to number of patients with the two biomarkers assessed.
