# Supplementary material for: Cilengitide in newly diagnosed glioblastoma: biomarker expression and outcome
Source: Oncotarget. 2016 Feb 22;7(12):15018–32. doi: 10.18632/oncotarget.7588 (PMC4924768; doi:10.18632/oncotarget.7588)
Supplement: Supplementary file 3 [file oncotarget-07-15018-s003.doc]

Supplementary Table 3. Association of integrin and pSMAD2 levels with PFS.

|  |  |  |  |  |
| --- | --- | --- | --- | --- |
|  |  |  |  |  |
|  | **Patients/events** | **Median (95% CI) in months** | **Hazard ratio (95% CI)** | **p value (score test)** |
| **CENTRIC** |  |  |  |  |
| **αvβ3, tumor cells** |  |  |  |  |
| < median | 214/144 | 13.0 (10, 15.4) | 1.0 | 0.56 |
| > median | 80/58 | 12.3 (7.8, 16.0) | 1.1 (0.8, 1.5) |  |
| **CORE** |  |  |  |  |
| **αvβ3, tumor cells** |  |  |  |  |
| < median | 147/123 | 6.0 (5.1, 7.0) | 1.0 | 0.52 |
| > median | 94/84 | 7.4 (5.9, 7.9) | 0.9 (0.7, 1.2) |  |
| **CENTRIC** |  |  |  |  |
| **αvβ3, endothelial cells** |  |  |  |  |
| < median | 141/99 | 10.6 (7.7, 16.0) | 1.0 | 0.68 |
| > median | 153/103 | 13.0 (10.6, 16.0) | 0.9 (0.7, 1.2) |  |
| **CORE** |  |  |  |  |
| **αvβ3, endothelial cells** |  |  |  |  |
| < median | 127/106 | 6.0 (4.4, 6.8) | 1.0 | 0.69 |
| > median | 114/101 | 7.7 (5.9, 7.8) | 1.0 (0.7, 1.2) |  |
| **CENTRIC** |  |  |  |  |
| **αvβ5, tumor cells** |  |  |  |  |
| < median | 144/94 | 12.0 (8.4, 16.0) | 1.0 | 0.50 |
| > median | 150/107 | 13.5 (10.6, 16.0) | 1.1 (0.8, 1.5) |  |
| **CORE** |  |  |  |  |
| **αvβ5, tumor cells** |  |  |  |  |
| < median | 127/105 | 6.7 (5.8, 7.8) | 1.0 | 0.55 |
| > median | 110/98 | 6.0 (5.2, 7.8) | 1.1 (0.8, 1.4) |  |
| **CENTRIC** |  |  |  |  |
| **αvβ5, endothelial cells** |  |  |  |  |
| < median | 145/98 | 10.8 (8.1, 13.5) | 1.0 | 1.0 |
| > median | 147/101 | 13.5 (10.5, 16.4) | 1.0 (0.8, 1.3) |  |
| **CORE** |  |  |  |  |
| **αvβ5, endothelial cells** |  |  |  |  |
| < median | 121/101 | 6.0 (5.1, 7.7) | 1.0 | 0.28 |
| > median | 115/101 | 6.9 (5.9, 7.8) | 0.9 (0.7, 1.1) |  |
| **CENTRIC** |  |  |  |  |
| **αvβ8, tumor cells** |  |  |  |  |
| < median | 139/92 | 13.1 (10.0, 16.4) | 1.0 | 0.71 |
| > median | 144/104 | 12.1 (8.1, 15.4) | 1.1 (0.8, 1.4) |  |
| **CORE** |  |  |  |  |
| **αvβ8, tumor cells** |  |  |  |  |
| < median | 115/94 | 6.1 (4.9, 7.8) | 1.0 | 0.85 |
| > median | 116/104 | 6.9 (5.9, 7.8) | 1.0 (0.8, 1.4) |  |
| **CENTRIC** |  |  |  |  |
| **αvβ8, endothelial cells** |  |  |  |  |
| < median | 258/181 | 12.2 (10.4, 13.6) | 1.0 | 0.57 |
| > median | 25/15 | 10.6 (6.0, 32.2) | 0.9 (0.5, 1.5) |  |
| **CORE** |  |  |  |  |
| **αvβ8, endothelial cells** |  |  |  |  |
| < median | 215/185 | 6.3 (5.9, 7.7) | 1.0 | 0.65 |
| > median | 16/13 | 6.1 (2.3, 9.2) | 1.1 (0.7, 2.00) |  |
| **CENTRIC** |  |  |  |  |
| **pSMAD2, tumor cells** |  |  |  |  |
| < median | 136/90 | 10.6 (7.7, 15.4) | 1.0 | 0.73 |
| > median | 145/103 | 13.3 (10.6, 16.0) | 1.0 (0.7, 1.3) |  |
| **CORE** |  |  |  |  |
| **pSMAD2, tumor cells** |  |  |  |  |
| < median | 113/95 | 7.5 (6.0, 7.8) | 1.0 | 0.12 |
| > median | 114/98 | 6.0 (4.2, 7.4) | 1.3 (0.9, 1.7) |  |
| **CENTRIC** |  |  |  |  |
| **pSMAD2, endothelial cells** |  |  |  |  |
| < median | 139/87 | 11.8 (7.8, 17.9) | 1.0 | 0.45 |
| > median | 142/106 | 12.1 (10.6, 14.4) | 1.1 (0.8, 1.5) |  |
| **CORE** |  |  |  |  |
| **pSMAD2, endothelial cells** |  |  |  |  |
| < median | 113/91 | 6.8 (5.9, 7.7) | 1.0 | 0.20 |
| > median | 114/102 | 6.1 (5.5, 7.8) | 1.2 (0.91, 1.6) |  |
