# Supplementary material for: Cilengitide in newly diagnosed glioblastoma: biomarker expression and outcome
Source: Oncotarget. 2016 Feb 22;7(12):15018–32. doi: 10.18632/oncotarget.7588 (PMC4924768; doi:10.18632/oncotarget.7588)
Supplement: Supplementary file 4 [file oncotarget-07-15018-s004.doc]

Supplementary Table 4. Association of integrin and pSMAD2 levels with OS.

|  |  |  |  |  |
| --- | --- | --- | --- | --- |
|  |  |  |  |  |
|  | **Patients/events** | **Median (95% CI) in months** | **Hazard ratio (95% CI)** | **p value (score test)** |
| **CENTRIC** |  |  |  |  |
| **αvβ3, tumor cells** |  |  |  |  |
| < median | 214/102 | 25.6 (23.4, 34.3) | 1.0 | 0.23 |
| > median | 80/44 | 23.6 (19.6, N) | 1.2 (0.9, 1.8) |  |
| **CORE** |  |  |  |  |
| **αvβ3, tumor cells** |  |  |  |  |
| < median | 147/111 | 14.1 (12.7, 15.6) | 1.0 | 0.37 |
| > median | 94/68 | 14.9 (12.9, 18.1) | 0.9 (0.6, 1.2) |  |
| **CENTRIC** |  |  |  |  |
| **αvβ3, endothelial cells** |  |  |  |  |
| < median | 141/66 | 30.0 (23.6, 35.1) | 1.0 | 0.36 |
| > median | 153/80 | 23.7 (21.7, 25.7) | 1.2 (0.8, 1.6) |  |
| **CORE** |  |  |  |  |
| **αvβ3, endothelial cells** |  |  |  |  |
| < median | 127/96 | 14.7 (13.1, 16.2) | 1.0 | 0.68 |
| > median | 114/83 | 14.3 (12.2, 16.9) | 0.9 (0.70, 1.3) |  |
| **CENTRIC** |  |  |  |  |
| **αvβ5, tumor cells** |  |  |  |  |
| < median | 144/73 | 24.3 (22.0, 31.0) | 1.0 | 0.39 |
| > median | 150/72 | 25.4 (22.9, N) | 0.9 (0.6, 1.2) |  |
| **CORE** |  |  |  |  |
| **αvβ5, tumor cells** |  |  |  |  |
| < median | 127/99 | 14.1 (12.7, 16.2) | 1.0 | 0.54 |
| > median | 110/79 | 14.2 (12.7, 16.1) | 0.9 (0.7, 1.2) |  |
| **CENTRIC** |  |  |  |  |
| **αvβ5, endothelial cells** |  |  |  |  |
| < median | 145/76 | 23.9 (21.6, 30.0) | 1.0 | 0.63 |
| > median | 147/69 | 25.7 (23.4, 35.1) | 0.9 (0.7, 1.3) |  |
| **CORE** |  |  |  |  |
| **αvβ5, endothelial cells** |  |  |  |  |
| < median | 121/93 | 14.1 (12.8, 15.6) | 1.0 | 0.37 |
| > median | 115/84 | 14.5 (12.4, 16.3) | 0.9 (0.7, 1.2) |  |
| **CENTRIC** |  |  |  |  |
| **αvβ8, tumor cells** |  |  |  |  |
| < median | 139/61 | 30.0 (24.3, N) | 1.0 | 0.06 |
| > median | 144/78 | 23.7 (20.3, 25.6) | 1.4 (1.0, 1.9) |  |
| **CORE** |  |  |  |  |
| **αvβ8, tumor cells** |  |  |  |  |
| < median | 115/83 | 14.0 (12.4, 15.4) | 1.0 | 0.82 |
| > median | 116/89 | 14.6 (13.1, 17.1) | 1.0 (0.7, 1.3) |  |
| **CENTRIC** |  |  |  |  |
| **αvβ8, endothelial cells** |  |  |  |  |
| < median | 258/127 | 25.4 (23.3, 30.9) | 1.0 | 0.93 |
| > median | 25/12 | 24.4 (15.3, N) | 1.0 (0.5, 1.8) |  |
| **CORE** |  |  |  |  |
| **αvβ8, endothelial cells** |  |  |  |  |
| < median | 215/160 | 14.3 (13.2, 15.6) | 1.00 | 0.16 |
| > median | 16/12 | 9.1 (628, 19.7) | 1.5 (0.9, 2.8) |  |
| **CENTRIC** |  |  |  |  |
| **pSMAD2, tumor cells** |  |  |  |  |
| < median | 136/61 | 25.9 (23.6, N) | 1.0 | 0.32 |
| > median | 145/77 | 23.9 (22.0, 31.0) | 1.2 (0.9, 1.7) |  |
| **CORE** |  |  |  |  |
| **pSMAD2, tumor cells** |  |  |  |  |
| < median | 113/83 | 14.7 (13.4, 16.5) | 1.0 | 0.42 |
| > median | 114/84 | 13.4 (12.6, 15.1) | 1.1 (0.8, 1.5) |  |
| **CENTRIC** |  |  |  |  |
| **pSMAD2, endothelial cells** |  |  |  |  |
| < median | 139/61 | 28.44 (23.9, N) | 1.0 | 0.18 |
| > median | 142/77 | 23.6 (21.1, 25.7) | 1.3 (0.90, 1.8) |  |
| **CORE** |  |  |  |  |
| **pSMAD2, endothelial cells** |  |  |  |  |
| < median | 113/80 | 14.3 (12.4, 17.0) | 1.0 | 0.28 |
| > median | 114/87 | 14.1 (12.9, 15.5) | 1.2 (0.9, 1.6) |  |
